# Anemia and the risk of Parkinson’s disease in Korean older adults: A nationwide population-based study

**DOI:** 10.1038/s41598-020-61153-5

**Published:** 2020-03-06

**Authors:** In Young Cho, Dong Wook Shin, Younjin Roh, Wooyoung Jang, Jin Whan Cho, Eun Ae Lee, Hyeonyoung Ko, Kyungdo Han, Jun Hyun Yoo

**Affiliations:** 1 Department of Family Medicine, Samsung Medical Center, Sungkyunkwan University School of Medicine, Seoul, 06351 Korea; 2 Department of Neurology, Gangneung Asan Hospital, University of Ulsan College of Medicine, Gangneung, 25440 Republic of Korea; 3 Department of Neurology, Samsung Medical Center, Sungkyunkwan University School of Medicine, Seoul, 06351 Korea; 4 Department of Family Medicine, Hansol Hospital, Seoul, 05616 Korea; 5 Department of Family Medicine, Kangbuk Samsung Hospital, Sungkyunkwan University School of Medicine, Seoul, 03181 Korea; 6 Department of Statistics and Actuarial Science, Soongsil University, Seoul, 06978 Korea

**Keywords:** Epidemiology, Parkinson's disease

## Abstract

Evidence of the association between anemia and risk of PD (Parkinson’s disease) have been accumulating. This study aimed to examine the relationship between anemia and risk of PD in Korean older adults. Korean adults aged 50 years or older who participated in the Korean National Screening Program (*n* = 12,342,278) between 2009 and 2013 were followed until 2015. Cox proportional hazards regression models were used to calculate the hazard ratio (HR) of PD, and participants were followed for a mean period of 5.0 years. At the end of follow-up, 3,844 adults were diagnosed with PD. After adjusting for potential confounders, participants with anemia had decreased risk of PD compared to adults without anemia (adjusted HR (aHR) 0.894, 95% CI: 0.809–0.989). Furthermore, aHR of PD was 0.698 (95% CI: 0.546–0.891) in moderate to severe anemia and 0.938 (95% CI: 0.843–1.044) in mild anemia. The protective effect of anemia was also more profound in men (aHR 0.888, 95% CI: 0.774–1.02) than in women (aHR 0.905, 95% CI: 0.782–1.048). In conclusion, anemia was associated with lower risk of PD, particularly for patients with moderate to severe anemia. Our study suggests that further studies may be needed to clarify the relationship between anemia and PD.

## Introduction

Parkinson’s disease (PD) is the second most common neurodegenerative disease worldwide^1,2^. The prevalence of PD was approximately 0.3% in the population aged over 40 years^1^. With increasing age, the prevalence of PD increases, and as the size of the aging population is rapidly increasing, the total number of PD patients is expected to increase over time^3^. The socioeconomic burden of PD is steadily increasing due to longevity and is estimated to be at least $14.4 billion a year in the United States^4,5^. The medical expense per patient was twice as high for PD patients than for other patients^5,6^.

No effective methods to prevent PD have been instituted since the pathogenesis of PD is not clearly understood. Moreover, results of previous studies concerning risk factors for PD have not been consistent. Therefore, further well-designed studies to reveal the risk factors for PD are a priority to prevent or delay development of PD. Some evidence suggest that anemia is potentially associated with development of PD, and results of previous studies concerning the relationship between anemia and PD have been controversial. A population-based cohort study using claims data in Taiwan demonstrated that newly diagnosed anemia increased the risk of PD^7^, and a previous case-control study in the U.S. showed that more patients in the PD group had anemia than patients in the control group^8^. However, another study found that increase in hemoglobin concentration in peripheral blood from <14 to ≥16 g/dL was associated with increased risk of PD in elderly men, and hemoglobin could serve as the most significant source of peripheral iron^9^. Concerning iron intake, one study reported that participants with high intake of iron had increased risk of PD, which is consistent with the idea that iron is a risk factor for PD^10^. However, a meta-analyses of 33 studies showed that iron levels in the blood and cerebrospinal fluid were not different between PD patients and healthy controls^11^.

To the best of our knowledge, a cohort study investigating the relationship between hemoglobin level and PD risk has not yet been conducted. Although case control studies studying this association have been carried out, there have been no cohort studies, especially in an Asian population^8^. In a recent cohort study conducted in Taiwan, anemia was not defined by hemoglobin levels but by diagnosis through claims data, which may create diagnostic limitations^7^. Anemia may have been under-diagnosed because hemoglobin levels of study subjects and severity of anemia were not examined. Also, well-known risk factors for PD, such as smoking and alcohol intake, were not adjusted for in the analysis.

Therefore, we aimed to study the relationship between hemoglobin level and development of PD in this longitudinal cohort study through a nationwide health screening program.

## Results

### Study participant characteristics

The demographics and comorbid diseases of study participants are shown in Table 1. Among the total of 2,241,119 participants, 3,844 were diagnosed with PD. In the PD patient group, the mean age was 64.75 ± 8.35 years. Patients with PD were older, had lower proportions of smokers and regular drinkers, and had a lower rate of regular physical activity. In addition, participants with PD had higher rates of hypertension and diabetes and had lower GFR than the non-PD group. The baseline characteristics of subjects with and without anemia are also provided in Supplementary Table S1.

**Table 1:** Baseline characteristics of study participants by presence of Parkinson’s disease.

	Total (*n* = 2,241,119)	Parkinson’s disease		*P* value
		No (*n* = 2,237,275)	Yes (*n* = 3,844)	
Age, years	57.41 ± 7.51	57.39 ± 7.50	64.75 ± 8.35	<0.0001
50–59	1,506,616	1,505,537 (67.29)	1,079 (28.07)	<0.0001
60–69	533,765	532,248 (23.79)	1,517 (39.46)	
≥70	200,738	199,490 (8.92)	1,248 (32.47)	
Sex				
Male	1,412,082	1,409,588 (63.00)	2,494 (64.88)	0.0161
Female	829,037	827,687 (37.00)	1,350 (35.12)	
Household income				
High	1,733,684	1,730,664 (77.36)	3,020 (78.56)	0.0737
Low	507,435	506,611 (22.64)	824 (21.44)	
Body mass index				
<18.5	881,893	880,394 (39.35)	1,499 (39.00	0.0077
18.5–25	618,575	617,582 (27.60)	993 (25.83)	
>25	740,651	739,299 (33.04)	1,352 (35.17)	
Smoking status				
Never smoker	1,216,804	1,214,324 (54.28)	2,480 (64.52)	<0.0001
Former smoker	441,611	440,780 (19.70)	831 (21.62)	
Current smoker	582,704	582,171 (26.02)	533 (13.87)	
Regular drinker	163,906	163,725 (7.32)	181 (4.71)	<0.0001
Regular exerciser	1,145,920	1,144,120 (51.14)	1,800 (46.83)	<0.0001
Hypertension	776,832	775,118 (34.65)	1,714 (44.59	<0.0001
Diabetes mellitus	272,880	272,249 (12.17)	631 (16.42)	<0.0001
Dyslipidemia	509,495	508,627 (22.73)	868 (22.58)	0.8204
Cancer	50,125	50,006 (2.24)	119 (3.10)	0.0003
Anemia	217,086	216,631 (9.68)	455 (11.84)	<0.0001
Anemia severity				
No anemia	2,024,033	2,020,644 (90.32)	3,389 (88.16)	<0.0001
Mild anemia	168,181	167,793 (7.50)	388 (10.09)	
Moderate-severe anemia	48,905	48,838 (2.18)	67 (1.74)	
Glomerular filtration, rate, ml/min2	86.79 ± 35.01	86.80 ± 35.02	82.99 ± 27.02	<0.0001
>60	2,129,951	2,126,488 (95.05)	3,463 (90.09)	<0.0001
30–60	111,168	110,787 (4.95)	381 (9.91)	

Data are presented as mean ± standard deviation for continuous variables.

Data are presented as number (percentage) for categorical variables.

### Association of anemia and PD

The enrolled study participants were followed for a mean period of 5.0 years. The incidence rates of PD in participants with anemia and without anemia were 0.328 and 0.411 per 1,000 person-years, respectively. The adjusted hazard ratio (aHR) of PD for the participants with anemia was 0.894 (95% confidence interval (CI): 0.809–0.989), shown in Table 2. Sensitivity analysis performed using the propensity score matching algorithm yielded similar results (overall aHR 0.891, 95% CI 0.798–0.996) (Supplementary Table S2). Competing risk analysis also showed similar results for PD risk in anemic subjects (Supplementary Table S3).

**Table 2:** Hazard ratio (HR) and 95% confidence intervals (CI) of Parkinson’s disease according to presence of anemia.

	Parkinson’s disease	Duration, person-year	Incidence rate^a^	Crude HR (95% CI)	Adjusted HR^b^ (95% CI)
**Total**					
Non-anemic	3,389	10,334,034.32	0.32795	1 (reference)	1 (reference)
Anemia	455	1,108,126.43	0.41060	1.247 (1.131–1.376)	0.894 (90.809–0.989)
**Male**					
Non-anemic	2,258	6,754,608.24	0.33429	1 (reference)	1 (reference)
Anemia	236	444,938.52	0.53041	1.571 (1.373–1.796)	0.888 (0.774–1.020)
**Female**					
Non-anemic	1,131	3,579,426.08	0.31597	1 (reference)	1 (reference)
Anemia	219	663,187.91	0.33022	1.046 (0.905–1.209)	0.905 (0.782–1.048)

^a^Incidence rates were calculated as number of diagnoses of PD per 1,000 person-years.

^b^Hazard ratios were adjusted for age, sex, household income, body mass index, smoking status, alcohol intake, physical exercise, hypertension, diabetes mellitus, dyslipidemia, cancer and glomerular filtration rate.

When analyzed by anemia severity, lower hemoglobin level was associated with lower risk of PD. The incidence rates of PD in participants with mild anemia and moderate to severe anemia were 0.446 and 0.281 per 1,000 person-years, respectively. The aHR was 0.938 (95% CI: 0.843–1.044) for the mild anemia group and 0.698 (95% CI: 0.546–0.891) for the moderate to severe anemia group (Table 3).

**Table 3:** Hazard ratio (HR) and 95% confidence interval (CI) of Parkinson’s disease according to severity of anemia.

	Parkinson’s disease	Duration, person-year	Incidence rate^a^	Crude HR (95% CI)	Adjusted HR^b^ (95% CI)
**Total**					
Non-anemic	3,389	10,334,034.32	0.32795	1 (reference)	1 (reference)
Mild anemia	388	869,541.92	0.44621	1.348 (1.214–1.498)	0.938 (0.843–1.044)
Moderate-severe anemia	67	238,584.50	0.28082	0.872 (0.684–1.110)	0.698 (0.546–0.891)
**Male**					
Non-anemic	2,258	6,754,608.24	0.33429	1 (reference)	1 (reference)
Anemia	212	402,056.30	0.52729	1.557 (1.352–1.792)	0.889 (0.769–1.027)
Moderate-severe anemia	24	42,882.22	0.55967	1.704 (1.140–2.548)	0.883 (0.589–1.323)
**Female**					
Non-anemic	1,131	3,579,426.08	0.31597	1 (reference)	1 (reference)
Anemia	176	467,485.62	0.37648	1.184 (1.010–1.388)	1.004 (0.855–1.179)
Moderate-severe anemia	43	195,702.29	0.21972	0.708 (0.522–0.960)	0.642 (0.472–0.872)

^a^Incidence rates were calculated as number of diagnoses of PD per 1,000 person-years.

^b^Hazard ratios were adjusted for age, sex, household income, body mass index, smoking status, alcohol intake, physical exercise, hypertension, diabetes mellitus, dyslipidemia, cancer and glomerular filtration rate.

The protective effect of anemia against PD was more prominent in men than in women (Table 2). The incidence rate of PD in men with anemia and without anemia was 0.334 and 0.530 per 1,000 person-years, respectively. The adjusted HR of PD in anemic men was 0.888 (95% CI: 0.774–1.02). In women, the incidence rate of PD was 0.316 for participants without anemia and 0.330 for those with anemia, with an aHR of 0.905 in anemic patients (95% CI: 0.782–1.048). A similar pattern was observed when data was analyzed according to severity of anemia in both sex groups (Table 3). In men, the aHR was 0.889 (95% CI: 0.769–1.027) for the mild anemia group and 0.883 (95% CI: 0.589–1.323) for the moderate to severe anemia group. In women, the aHR was 1.004 (95% CI: 0.855–1.179) for the mild anemia group and 0.642 (95% CI: 0.472–0.872) for the moderate to severe anemia group (Table 3). Other factors associated with risk of PD were older age, male sex, smoking, and alcohol consumption (Table 4).

**Table 4:** Subgroup analysis of hazard ratios (HR) of Parkinson’s disease.

	Unadjusted HR (95% CI)	Adjusted HR (95% CI)
Anemia	1.247 (1.131–1.376)	0.949 (0.858–1.049)
Age, years		
50–59	1 (reference)	1 (reference)
60–69	3.537 (3.271–3.824)	3.332 (3.077–3.608)
≥70	7.995 (7.368–8.674)	7.196 (6.601–7.845)
Sex		
Male	1 (reference)	1 (reference)
Female	0.919 (0.860–0.982)	0.697 (0.644–0.755)
Income		
High	1 (reference)	1 (reference)
Low	0.921 (0.852–0.994)	0.920 (0.852–0.994)
Body mass index		
<18.5	1.079 (0.995–1.169)	1.060 (0.977–1.150)
18.5–25	1 (reference)	1 (reference)
>25	1.140 (1.051–1.238)	1.123 (1.034–1.220)
Smoking status		
Never smoker	1 (reference)	1 (reference)
Former smoker	0.934 (0.864–1.011)	0.816 (0.747–0.892)
Current smoker	0.469 (0.427–0.515)	0.479 (0.432–0.530)
Regular drinker	0.639 (0.550–0.742)	0.762 (0.654–0.887)
Regular exerciser	0.839 (0.787–0.894)	0.948 (0.888–1.011)
Hypertension	1.473 (1.383–1.570)	1.002 (0.936–1.072)
Diabetes mellitus	1.425 (1.308–1.552)	1.193 (1.092–1.302)
Dyslipidemia	0.997 (0.925–1.076)	0.945 (0.874–1.021)
Cancer	1.526 (1.271–1.832)	1.132 (0.943–1.360)
Glomerular filtration rate, ml/min2		
>60	1 (reference)	1 (reference)
30–60	1.983 (1.784–2.204)	1.133 (1.016–1.262)

CI confidence interval

For anemic patients, non-anemic study participants were used as reference group.

For regular drinkers, non-regular drinkers were used as reference group.

For hypertensive study participants, non-hypertensive participants were used as reference group.

For diabetic study participants, non-diabetic participants were used as reference group.

For dyslipidemic study participants, non-dyslipidemic participants were used as reference group.

For cancer study participants, non-cancer participants were used as reference group.

## Discussion

This is the first study to investigate the relationship between hemoglobin level and risk of PD in the Korean population. We found that low hemoglobin level was negatively associated with risk of developing PD, especially in male and moderate-to-severe anemia groups. The strengths of this study include: 1) use of a large sample, representing the general population, which identified a sufficient number of patients with PD and 2) use of the definition of anemia based on hemoglobin level.

The mechanisms underlying the association between anemia and PD is expected to be complicated and not fully understood. The neuronal degeneration in PD is mediated by either apoptosis or necrosis^12^. Neurons in the substantia nigra tend to undergo apoptosis more frequently in the brains of PD patients than in normal brains^13^. While the exact mechanism of the degeneration of dopaminergic neurons in PD is not fully elucidated, several possible mechanisms suggesting anemia as a protective factor against PD can be proposed. First, oxidative stress and iron metabolism are hypothesized as possible mechanisms leading to dopaminergic neuronal death in the CNS^14,15^. Iron is the most abundant metal in the adult brain^16^, and it accumulates in neurons of the substantia nigra in PD patients^17,18^. Iron is involved in the reactive oxygen species (ROS) system, leading to free radical and oxidative damage to neurons^19–21^. This explanation postulates that overproduced reactive oxygen/nitrogen species can cause neurodegeneration^20,21^. Free radicals attack cell membrane lipids, leading to lipid peroxidation, which has been found to be increased in both the substantia nigra and red blood cells of PD patients^22^. Reactive species act as the catalyst for mitochondrial dysfunction, as well as alpha-synuclein misfolding, aggregation, and accumulation^23,24^. Moreover, iron itself can cause toxicity to neurons and may be the key factor for the association of anemia and PD. CSF ferritin level was elevated in PD patients and decreased upon treatment with an iron chelator, improving symptoms of PD^22^. In addition, evidence suggests that free iron boosts alpha synuclein aggregation in the substantia nigra^25,26^. Therefore, this study supports the hypothesis that elevated iron level is toxic to neurons in the substantia nigra.

Also, alpha-synuclein, one of the most important proteins in the pathogenesis of PD, may be associated with anemia. An insoluble aggregate of alpha-synuclein is a major component of the Lewy body, an intracellular inclusion that is the hallmark of PD^27^. More than 99% of alpha-synuclein in human blood exists inside the blood cells, mostly in red blood cells; but alpha-synuclein’s functions in hematopoiesis and immune reactions are unknown^28,29^. Erythrocytes secrete alpha-synuclein into plasma, but the significance of this is unknown. The total amount of alpha-synuclein in blood plasma in PD patients was not different from that of those without PD^30^. The distribution of alpha-synuclein in red blood cells in PD patients is different from control groups^31^. Although the molecular mechanism of alpha-synuclein regulation in blood cells and neurons is not known, anemia and PD may be associated by alpha-synuclein. Decreased alpha-synuclein in the red blood cells of anemic subjects may result in decreased secreted alpha-synuclein; therefore the amount of alpha-synuclein undergoing pathologic change will be decreased^30^. Further study is needed to investigate the roles of alpha-synuclein in anemia and PD.

One notable finding was the sex difference in the association between anemia and PD, with stronger association in males than females. Some epidemiologic studies were conducted to investigate the difference in incidence or prevalence of PD between sexes, and men had higher incidence and prevalence of PD than women^2,32^. In our study, low hemoglobin level decreased the risk of PD to a larger degree in men than in women. Iron may play a more important role in the pathogenesis of PD in men, or men may be more vulnerable to iron accumulation in the substantia nigra than women. More research on the etiology of PD is needed to clarify the reasons why (1) PD is more frequent in men and (2) anemia lowers the risk of PD more in men than in women.

Our study is contrary to several previous studies studying the association between anemia and PD^8,33^, including a recent large-scale study in Taiwan, which showed a positive association between diagnosis of iron-deficiency anemia (IDA) and PD^7^. In the Taiwanese study, the claim code of IDA, iron supplementation therapy, was used as exposure. Iron supplementation itself or changes in iron level may have contributed to the development of PD in this population. In addition, unlike the mentioned study, our study accessed demographic and social information of subjects and adjusted for well-known risk factors, such as alcohol consumption and smoking, which were not adjusted for in the mentioned study.

Interestingly, our study found that smoking and drinking were associated with lower risk of PD. However, there is numerous evidence on the inverse association between smoking and PD, which is stronger for current than former smokers compared to non-smokers^34,35^. A European population-based prospective cohort study also demonstrated that current smokers had lower risk of PD than former smokers compared to non-smokers, and a strong dose-response relationship was also found^36^. Substances in tobacco such as 2,3,6-trimethyl-1,4-naphthoquinone and nicotine itself have been suggested to affect dopamine metabolism and activity in the brain^37,38^. Meanwhile, previous research on alcohol and PD risk have been inconsistent. However, a recent meta-analysis suggested an inverse association between drinking and PD, which was supported by case-control studies but not clear in prospective studies^39^. Another meta-analysis of longitudinal studies also suggested lower PD risk in drinkers compared with non-drinkers^40^, which was thought to be consistent with the elevating effects of alcohol on urate levels^41,42^.

Our study has several limitations. The level of hemoglobin at the time of NHSP examination was used to assess the presence and severity of anemia, and we could not eliminate the factor of iron replacement at the time of examination. For this reason, some of the participants who temporarily had normal hemoglobin level could have been misclassified into the non-anemic group. Thus, the incidence of PD in the non-anemic group may have been underestimated. Moreover, we were unable to clarify the effect of iron replacement therapy itself on the risk of PD. Another limitation is that we were unable to define the physiological onset of anemia, because hemoglobin levels were defined only at inclusion of the study. Because the reliability of PD diagnosis was also one of our concerns, we used the registration of the NHIS, which needs confirmation of healthcare providers to verify the validity of diagnosis. Due to strict classification of PD patients, those who were not registered in this system may have been misclassified to the control group. The probability of misclassification was the same in both groups, which may have led to underestimation of the protective effect of low hemoglobin levels. Another possible limitation is reverse causation. However, results in our sensitivity analysis of 2-year lag time were consistent with our main findings (Supplementary Tables S4 and S5). Finally, this study lacks information on some confounding factors that may affect the incidence of PD. These include occupational history, coffee or caffeine intake, history of exposure to toxic molecules including pesticides, living environments, and the use of well water, which we could not adjust for in this study.

In conclusion, we demonstrated that lower hemoglobin levels decreased the risk of PD. The protective effect of anemia was stronger in men than in women. The association of anemia and development of PD could be explained by oxidative stress, alpha-synucleinopathy, and dysregulation of iron homeostasis. Further study is needed to clarify the mechanisms underlying the association of anemia and risk of PD.

## Methods

### Study design and participants

The Korean National Health Insurance (KNHI) is a compulsory insurance program that covers most of the healthcare services conducted in Korea. KNHI also operates a National Health Screening Program (NHSP) for members aged 40 years and older. All members undergoing the NHSP are required to complete a self-reported questionnaire and routine examinations, including blood test and chest X-ray^43^.

This study used National Health Insurance Service (NHIS) data. We extracted medical claims and demographic information from the NHIS data, including age, sex, monthly insurance premium (proxy for household income level), and disease codes using the medical diagnosis of International Classification of Disease, 10^th^ revision, ICD-10. We also extracted health screening data, including lifestyle information (smoking, alcohol consumption, and physical activity), laboratory test results, and physical exam information such as weight and height.

**Figure 1:**
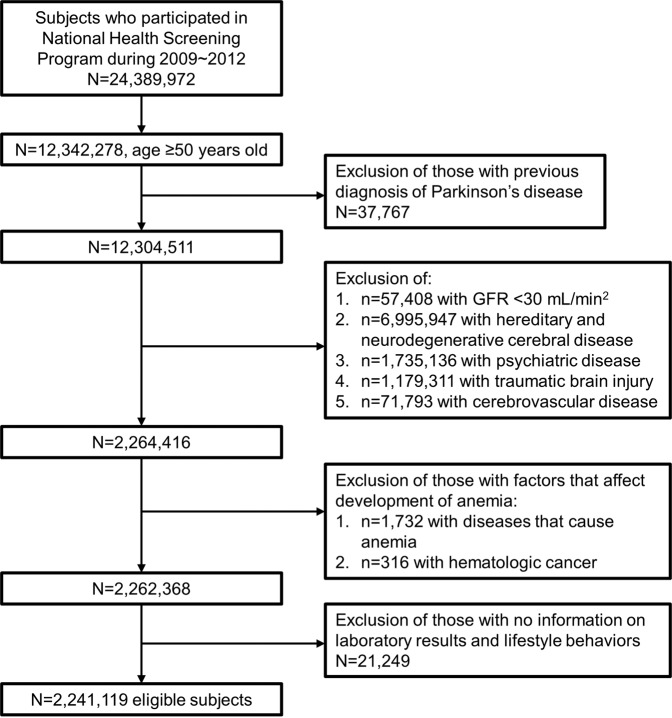
Flowchart of the subject selection process.

This retrospective cohort study was conducted using data collected between 2009 and 2015. We used data beginning in 2009 because serum creatinine level was first included in routine blood tests of the NHSP at that time. The study cohort consisted of all Korean adults aged 50 years or older who had undergone the NHSP (*n* = 12,342,278) between 2009 and 2013. Participants previously diagnosed with PD (*n* = 37,767) and those who had glomerular filtration rate (GFR) less than 30 mL/min (*n* = 57,408) were excluded. Because the objective of this study was to clarify the relationship between anemia, especially iron-related anemia, and PD, we excluded participants whose decreased renal function may affect both iron-metabolism and development of PD^44–46^. Study participants with any diseases or medical conditions that increase PD risk were further excluded. We excluded participants who had (1) hereditary and degenerative disease of the central nervous system other than PD (ICD-10: G10~G14, G23~G26, G30~G32, G35~G37, G40~G47, G50~G59, G60~G64, G70~G73, G80~G83, and G90~G99) (*n* = 6,995,947), (2) psychiatric disorders (ICD-10: F00~F99) (*n* = 1,735,136), (3) traumatic brain injury (ICD-10: S02, S06, and S07~S09) (*n* = 1,179,311), and (4) cerebrovascular diseases (ICD-10: I60~I69) (*n* = 71,793). Participants with diseases or medical conditions likely to cause anemia were also excluded. Participants who were diagnosed with aplastic anemia or hemolytic anemia (ICD-10: D55~D59, D61) (*n* = 1,732), hematologic malignancy, or other blood disorders (ICD-10: C81~C85, C88, C90~C96, D46, D70~D77) (*n* = 316) were excluded. Finally, study participants with missing data were excluded. As a result, a total of 2,241,119 Korean adults were included in this study (Fig. 1).

The Institutional Review Board (IRB) of Samsung Medical Center approved this study (IRB file No. 2017-05-142) and waived the need for informed consent from the study participants as part of the study approval, because of the anonymity of the data retrieved from the NHIS database. All study methods were performed according to the guidelines approved by the Institutional Review Board of Samsung Medical Center, in accordance with the ethical standards in the 1964 Declaration of Helsinki and its later amendments.

### Definition of anemia

Anemia was defined through a one-time result obtained at the baseline health screening examinations. We used the anemia definition of the World Health Organization (WHO): hemoglobin level <13 g/dL for men and <12 g/dL for women. Anemia was categorized by severity as mild (Hb ≥ 11 g/dL) and moderate to severe (Hb < 11 g/dL).

### Main outcome

The main outcome of this study was PD incidence. We used ICD-10 codes to identify diagnosis of PD through diagnosis in medical expense claims (ICD-10 code G20) for both outpatient visits and hospitalizations. To ensure the validity of PD diagnosis, we used also used the registry system for rare intractable diseases (RIDs) in the KNHI. Enrollment in this system reduces patient expenses when requiring long-term and expensive treatment, and registration requires confirmation of diagnosis by a physician. Patients with proven PD diagnosis can be registered in this system, and usually neurologists or primary care physicians who have examined the patient issue the required certification of PD diagnosis. Therefore, we identified new PD onset through claims data with ICD-10 code for PD (G20) confirmed by the RID registration code (V124). This method has also been used in previous epidemiologic studies on PD based on the Korean population, and is regarded to be reasonably accurate^47^. Follow-up time was calculated as time from beginning of the study to the diagnosis of PD, death, or the final day of study.

### Measurements

Physical measurements and serum biochemical parameters were measured by trained staff through standardized protocols. GFR was calculated via the Modification of Diet in Renal Disease (MDRD) equation. Body mass index (BMI) was calculated as weight divided by squared height (kg/m2) and was categorized into three groups according to Asian obesity criteria: underweight (BMI < 18.5 kg/m2), normal to overweight (BMI = 18.5 to 25 kg/m2), and obese (BMI ≥ 25 kg/m2)^48^. Household income status was divided into two groups of either high or low based on insurance premiums. Smoking status was categorized into never, former, or current smoker. Both alcohol intake and physical activity were classified into non-regular and regular groups. Comorbid conditions that may affect anemia or PD were considered and identified using ICD-10 codes: hypertension (ICD-10: I10-I15), diabetes mellitus (ICD-10: E10-E14), dyslipidemia (ICD-10: E78), and cancer (ICD-10: C code, D00-D09).

### Statistical analyses

Chi-square test was performed to compare the baseline characteristics between study participants with or without PD, and with or without anemia. The incidence rate of PD was calculated as number of diagnoses of PD per 1000 person-years with 95% confidence intervals (95% CIs). The Cox proportional hazards model was used to measure the risk of PD according to the presence or severity of anemia. Cox proportional models were adjusted for age, sex, BMI, alcohol consumption, smoking, physical activity, household income status, hypertension, diabetes, dyslipidemia, GFR, and cancer. We conducted sensitivity analysis by performing 1:2 propensity score matching to adjust for the baseline differences, and additionally calculated PD risk for the anemic and non-anemic groups. We also conducted sensitivity analysis for the main outcomes by giving a lag time of 2 years to account for reverse causation. We also used the Fine and Gray competing risk model to account for the risk of death, which is assumed to be different between the groups with and without anemia. All statistical analyses were performed using STATA 14.0 (Stata Corp, Texas, USA), with statistical significance defined as two-tailed p-value <0.05.

## Supplementary information

Supplementary Information.

## Data Availability

The datasets generated during and/or analyzed during the current study are available from the corresponding author on reasonable request.
