# Supplementary material for: Anemia and the risk of Parkinson’s disease in Korean older adults: A nationwide population-based study
Source: Sci Rep. 2020 Mar 6;10:4268. doi: 10.1038/s41598-020-61153-5 (PMC7060334; doi:10.1038/s41598-020-61153-5)
Supplement: Supplementary file 1 — Supplementary Information. [file 41598_2020_61153_MOESM1_ESM.docx]

**Supplementary Information**

**Title of manuscript: Anemia and the risk of Parkinson’s disease in Korean older adults: A nationwide population-based study**

**Author list:**

In Young Cho, Dong Wook Shin, Younjin Roh, Wooyoung Jang, Jin Whan Cho, Eun Ae Lee, Hyeonyoung Ko, Kyungdo Han, Jun Hyun Yoo

**Supplementary Table S1.** Baseline characteristics of study participants by presence of anemia.

|  | Anemia | | *P* value |
| --- | --- | --- | --- |
|  | No | Yes |  |
|  | (*n* = 2,024,033) | (*n* = 217,086) |  |
| Age, years | 57.17 ± 7.24 | 59.6 ± 9.37 | <0.0001 |
| 50-59 | 1,382,547 (68.31) | 124,069 (57.15) | <0.0001 |
| 60-69 | 479,609 (23.7) | 54,156 (24.95) |  |
| ≥ 70 | 161,877 (8.00) | 38,861 (17.90) |  |
| Sex |  |  | <0.0001 |
| Male | 1,324,958 (65.46) | 87,124 (40.13) |  |
| Female | 699,075 (34.54) | 129,962 (59.87) |  |
| Household income |  |  | <0.0001 |
| High | 1,574,208 (77.78) | 159,476 (73.46) |  |
| Low | 449,825 (22.22) | 57,610 (26.54) |  |
| Body mass index |  |  | <0.0001 |
| < 18.5 | 766,803 (37.88) | 115,090 (53.02) |  |
| 18.5-25 | 566,506 (27.99) | 52,069 (23.99) |  |
| > 25 | 690,724 (34.13) | 49,927 (23.00) |  |
| Smoking status |  |  | <0.0001 |
| Never smoker | 1,060,301 (52.39) | 156,503 (72.09) |  |
| Former smoker | 413,726 (20.44) | 27,885 (12.85) |  |
| Current smoker | 550,006 (27.17) | 32,698 (15.06) |  |
| Regular drinker | 154,876 (7.65) | 9,030 (4.16) | <0.0001 |
| Regular exerciser | 1,049,257 (51.84) | 96,663 (44.53) | <0.0001 |
| Hypertension | 704,394 (34.8) | 72,438 (33.37) | <0.0001 |
| Diabetes mellitus | 247,706 (12.24) | 25,174 (11.6) | <0.0001 |
| Dyslipidemia | 468,511 (23.15) | 40,984 (18.88) | <0.0001 |
| Cancer | 40,515 (2.00) | 9,610 (4.43) | <0.0001 |
| Glomerular filtration, rate, ml/min^2^ | 86.84 ± 35.37 | 86.29 ± 31.37 | <0.0001 |
| > 60 | 1,931,759 (95.44) | 198,192 (91.30) | <0.0001 |
| 30-60 | 92,274 (4.56) | 18,894 (8.70) |  |

Data are presented as mean ± standard deviation for continuous variables.

Data are presented as number (percentage) for categorical variables.

**Supplementary Table S2.** Hazard ratio (HR) and 95% confidence intervals (CI) of Parkinson’s disease (PD) according to presence of anemia, after propensity score matching.

|  | **Parkinson’s disease** | **Duration, person-year** | | **Incidence rate^a^** | | **Crude HR (95% CI)** | |  |
| --- | --- | --- | --- | --- | --- | --- | --- | --- |
| **Total** | | |  | |  | |  | |
| Non-anemic | 1,014 | 2,210,673.09 | | 0.45868 | | 1 (reference) | |  |
| Anemia | 452 | 1,103,318.49 | | 0.40967 | | 0.891 (0.798-0.996) | |  |
| **Male** | | |  | |  | |  | |
| Non-anemic | 541 | 880,063.89 | | 0.61473 | | 1 (reference) | |  |
| Anemia | 236 | 444,919.63 | | 0.53043 | | 0.863 (0.741-1.006) | |  |
| **Female** | | |  | |  | |  | |
| Non-anemic | 473 | 1,330,609.2 | | 0.35548 | | 1 (reference) | |  |
| Anemia | 216 | 658,398.86 | | 0.32807 | | 0.92 (0.783-1.080) | |  |

Propensity score matching (1:2) was performed based on age, sex, household income, body mass index, smoking status, alcohol intake, physical exercise, hypertension, diabetes mellitus, dyslipidemia, cancer and glomerular filtration rate.

^a^Incidence rates were calculated as number of diagnoses of PD per 1,000 person-years.

**Supplementary Table S3.** Results of competing risk analysis.

|  | **SHR (95% CI)** |
| --- | --- |
| **Total** |  |
| Anemia | 0.932 (0.824-1.055) |
| **Male** |  |
| Anemia | 0.884 (0.749-1.043) |
| **Female** |  |
| Anemia | 0.988 (0.821-1.189) |

SH, subdistribution hazard ratio; CI, confidence intervals

Fine and Gray’s competing risk model considering death as competing event was used, adjusting for age, sex, household income, body mass index, smoking status, alcohol intake, physical exercise, hypertension, diabetes mellitus, dyslipidemia, cancer and glomerular filtration rate.

**Supplementary Table S4.** Hazard ratio (HR) and 95% confidence intervals (CI) of Parkinson’s disease according to presence of anemia, after 2 years of lag time.

|  | **Parkinson’s disease** | **Duration,**  **person-year** | **Incidence rate** ^a^ | **Crude HR**  **(95% CI)** | **Adjusted HR^b^**  **(95% CI)** |
| --- | --- | --- | --- | --- | --- |
| **Total** | |  |  |  |  |
| Non-anemic | 2593 | 10,313,842.33 | 0.25141 | 1 (reference) | 1 (reference) |
| Anemia | 339 | 1,102,978.85 | 0.30735 | 1.211 (1.081-1.356) | 0.893 (0.795-1.003) |
| **Male** | |  |  |  |  |
| Non-anemic | 1740 | 6,738,608.15 | 0.25821 | 1 (reference) | 1 (reference) |
| Anemia | 173 | 441,234.17 | 0.39208 | 1.483 (1.268-1.734) | 0.87 (0.741-1.022) |
| **Female** | |  |  |  |  |
| Non-anemic | 853 | 3,575,234.18 | 0.23859 | 1 (reference) | 1 (reference) |
| Anemia | 166 | 661,744.68 | 0.25085 | 1.052 (0.891-1.243) | 0.92 (0.777-1.089) |

^a^Incidence rates were calculated as number of diagnoses of PD per 1,000 person-years.

^b^Hazard ratios were adjusted for age, sex, household income, body mass index, smoking status, alcohol intake, physical exercise, hypertension, diabetes mellitus, dyslipidemia, cancer and glomerular filtration rate.

**Supplementary Table S5.** Hazard ratio (HR) and 95% confidence interval (CI) of Parkinson’s disease according to severity of anemia, after 2 years of lag time.

|  | **Parkinson’s disease** | **Duration,**  **person-year** | **Incidence rate^a^** | **Crude HR**  **(95% CI)** | **Adjusted HR^b^**  **(95% CI)** |
| --- | --- | --- | --- | --- | --- |
| **Total** | |  |  |  |  |
| Non-anemic | 2,593 | 10,313,842.33 | 0.25141 | 1 (reference) | 1 (reference) |
| Mild anemia | 292 | 865,890.85 | 0.33722 | 1.315 (1.165-1.484) | 0.94 (0.831-1.064) |
| Moderate-severe anemia | 47 | 237,088.00 | 0.19824 | 0.813 (0.610-1.085) | 0.674 (0.50-0.902) |
| **Male** | |  |  |  |  |
| Non-anemic | 1,740 | 6,738,608.15 | 0.25821 | 1 (reference) | 1 (reference) |
| Anemia | 156 | 399,205.29 | 0.39078 | 1.471 (1.249-1.733) | 0.87 (0.736-1.029) |
| Moderate-severe anemia | 17 | 42,028.88 | 0.40448 | 1.598 (0.991-2.576) | 0.87 (0.538-1.406) |
| **Female** | |  |  |  |  |
| Non-anemic | 853 | 3,575,234.18 | 0.23859 | 1 (reference) | 1 (reference) |
| Anemia | 136 | 466,685.56 | 0.29142 | 1.205 (1.006-1.444) | 1.034 (0.86-1.242) |
| Moderate-severe anemia | 30 | 195,059.12 | 0.15380 | 0.666 (0.463-0.959) | 0.61 (0.423-0.88) |

^a^Incidence rates were calculated as number of diagnoses of PD per 1,000 person-years.

^b^Hazard ratios were adjusted for age, sex, household income, body mass index, smoking status, alcohol intake, physical exercise, hypertension, diabetes mellitus, dyslipidemia, cancer and glomerular filtration rate.
